# The effects of visual half-field priming on the categorization of familiar intransitive gestures, tool use pantomimes, and meaningless hand movements

**DOI:** 10.3389/fpsyg.2014.00454

**Published:** 2014-05-27

**Authors:** Honorata Helon, Gregory Króliczak

**Affiliations:** Action and Cognition Laboratory, Department of Social Sciences, Institute of Psychology, Adam Mickiewicz University in PoznańPoznań, Poland

**Keywords:** intransitive gestures, tool use pantomimes, meaningless actions, categorization, priming, pictures, words, representation

## Abstract

Although the control of meaningful gestures is one of the most left-lateralized functions, the relative contribution of the two hemispheres to their processing is still debated. We tested the effects of primes appearing in the left or right visual field in the form of pictures (Experiment 1), and words (Experiment 2) on categorization of movies showing intransitive (“communicative”) gestures, tool use (transitive) pantomimes, and meaningless movements. Fifteen participants (eight women) watched 36 movies (12 from each category) primed for 150 ms with either a congruent or incongruent stimulus followed by a 50-ms mask. On congruent trials, a picture or word was directly related to the presented gesture, including nonsense pictures or non-words for meaningless actions. On incongruent trials, a picture or word belonged to a different category. In Experiment 1, intransitive gestures were categorized significantly faster than the other two types of hand movements. Moreover, whereas the categorization of transitive gestures was significantly facilitated by congruent pictures on the right, the effect was weaker for intransitive, and reversed for meaningless movements. In Experiment 2, intransitive gestures were again categorized significantly faster, but transitive significantly slower than the other two gesture categories. Yet, there was now a significant facilitation of intransitive, and inhibition of transitive gesture categorization following congruent prime words in the right visual field, and significantly faster categorization of intransitive gestures following incongruent words in the left visual field. These outcomes lend support to the complexity account of differences in left-hemisphere representations of meaningful gestures reported in the neuropsychological, behavioral, and neuroimaging literature. Nevertheless, they also indicate that the representations of intransitive gestures show some differential, and sometimes counterintuitive sensitivity to right hemisphere processing.

## Introduction

Our current knowledge on the laterality of representations underlying meaningful gestures comes primarily from research on patients with acquired brain injuries, and more recently from experiments using functional neuroimaging (for reviews, see Frey, 2008; Rumiati et al., 2010; Goldenberg, 2013a; see also Goldenberg, 2013b; Króliczak, 2013a). These studies overwhelmingly point to the left hemisphere as the seat of the control of gesture. Yet, in the majority of these projects the emphasis was put primarily on manual performance, e.g., the planning and subsequent execution of conventionalized hand movements based on verbal commands, or the quality of imitation of the just seen meaningful vs. meaningless actions. Therefore, substantially less is known about the laterality of neural mechanisms involved in recognition, or even a potentially easier process of the categorization of skilled gestures (cf. Rumiati et al., 2010; pp. 224–225).

Among the gestures whose specific representations have been extensively explored and debated are sequential hand movements and/or postures frequently used in everyday communication, which are referred to as *intransitive gestures* (e.g., waving goodbye, beckoning, or hitchhiking), and less frequently used object-related *transitive actions* often referred to as *tool use pantomimes* (e.g., simulated use of a hammer, scissors, or a key). Of course, counter to transitive or tool use actions, the former gestures do not require real or imagined objects to convey their meaning. Yet despite these differences, empirical evidence in favor of independent (dissociable) mechanisms for the two types of gestures is inconclusive. On the one hand, patients with apraxia have been found either less impaired during performance of familiar intransitive gestures (Roy et al., 1991; Foundas et al., 1999; Haaland et al., 2000; cf. Mozaz et al., 2002) or not affected at all following left hemisphere lesions, despite showing considerable impairments during tool use pantomime (Rapcsak et al., 1993; Dumont et al., 1999, see also Stamenova et al., 2010). At first glance, then, such neuropsychological data suggest that the neural representations of transitive skills are lateralized more to the left hemisphere. Alternatively, the left hemisphere may support independent mechanisms for transitive and intransitive skills, with a possible extension of the processing of the latter to the right hemisphere. On the other hand, recent neuroimaging and behavioral evidence (Carmo and Rumiati, 2009; Kroliczak and Frey, 2009; see also Mozaz et al., 2009; Króliczak, 2013b) indicates that tool use pantomime and imitation may simply place higher demands on a common representational system mediating both intransitive and transitive manual skills, a system with close ties to language functions (Kroliczak et al., 2011; for a review, see Króliczak, 2013a).

Whether independent, for example differently lateralized, or rather common mechanisms are also involved in the visual processing (i.e., perception or recognition) of the two gesture categories is even more inconclusive. The reports from recent functional neuroimaging studies that directly addressed this question (Villarreal et al., 2008; Króliczak, 2013b) indicate that bilateral networks of areas are engaged during watching of both intransitive and transitive actions. The most striking difference was such that in the former project a greater involvement for perception of intransitive gestures was observed in a left-hemisphere structure located within a common network, specifically in the left inferior frontal gyrus (which was also engaged by transitive gestures but to a lesser degree). In the latter project, conversely, a greater and some bilateral involvement was observed for the perception of transitive actions, and only in areas that were outside of the common network of activation mediating both gesture categories (which may, arguably, reflect some dissociable mechanisms). Furthermore, whereas Villarreal et al. (2008) linked the greater left inferior frontal activity to the recognition of semantic content conveyed by their symbolic intransitive gestures, Króliczak (2013b) linked the observed increases of activity to the need for deeper visual encoding and more complex visuo-spatial transformations required for the processing of transitive (tool use) pantomimes. A preliminary conclusion that can be drawn from these two studies is such that the mechanisms mediating gesture categorization and/or recognition might be organized and/or lateralized somewhat differently from the mechanisms underlying their skilled performance.

In order to shed some new light on the above-mentioned controversies and arguments, in this study we tested (1) whether or not the potential differences in the familiarity and/or complexity between intransitive and transitive gestures are also evident in accuracy and response times accompanying their categorization, (2) whether or not the representations of the two gesture categories show different sensitivity to lateralized visual and linguistic cues, and if there are no clear hints of dissociable mechanisms, (3) whether or not the categorization of meaningful gestures differs from that of the processing of meaningless actions (that we used in this study as a control condition).

If intransitive gestures are indeed easier to categorize, this should be reflected at least in shorter response times. Moreover, if representations of the two gesture categories show different sensitivity to visual and semantic cues, and/or they are differently organized in the brain, we should observe distinct effects of such cues on their categorization (e.g., facilitation or inhibition of response times), possibly modulated by the side where the cues are presented. In particular, whereas congruent cues were expected to facilitate categorization mainly when projected first to the hemisphere specialized in processing of a particular gesture type, incongruent cues were expected to interfere most when projected to this same hemisphere, and their influence could be much weaker—but perhaps comparable with congruent cues—when reaching the less specialized hemisphere first. Finally, we also expected that the pattern of response times for the categorization of meaningless hand movements, that have no prior representation in the brain, would not resemble the patterns observed for meaningful gestures.

## Experiments

Although the order of the two experiments described here—one with pictures, and one with words as primes—was counterbalanced across the whole sample of subjects, for simplicity we will nevertheless refer to the use of pictures as Experiment 1, and the use of words as Experiment 2. The two experiments were carried out in *Action and Cognition Laboratory* in the Institute of Psychology at Adam Mickiewicz University in Poznań, Poland. Participants took no longer than 22 min to complete the whole study. Approved by the local Ethics Committee, this research was performed in accordance with principles of the Helsinki 1964 Declaration.

Seventeen volunteers partook in this research after giving their informed consent. The results from two participants (two women) were excluded from further analyses because of low accuracy (56.1%, with the average accuracy of 82% and *SD* = 8.5 in both studies) or the low number of the recorded responses (2%) due to an equipment malfunction.

### Experiment 1: categorization of gestures primed by pictures

#### Methods

All 15 healthy volunteers (eight women, mean age = 23.0; *SD* = 1.5) who contributed to this research had normal or corrected-to-normal visual acuity, and were native speakers of Polish. Although handedness was not measured with a questionnaire, before the study participants explicitly declared which hand they typically use in daily activities such as writing, throwing, and using a spoon. The vast majority of subjects (13) were right-handed. (The RT patterns of two left-handed individuals were indistinguishable from those of right-handers, most likely because similarly to the majority of left-handers they had praxis and language typically lateralized; see Kroliczak et al., 2011).

Before the experiment proper, subjects participated in a short pre-training phase composed of trials containing two movies from each of the to-be-tested category. Although the trial structure was the same, the videos used were recorded on a different occasion with a different background. It was during this introduction to the study that participants were asked for the first time to fixate the cross in the middle of the screen throughout the study. As confirmed by the experimenter, they were indeed able to maintain fixation when the priming pictures were shown, which is critical for this paradigm.

Participants were seated 57 cm in front of the computer monitor, which subtended the visual angle of 30 × 18.5°. Thirty six centrally presented short videos showing gestures performed by an actor were used as target stimuli. Only the right arm and hand, chest and the right upper leg were visible on the screen, whereas the face and most of the left side of the body remained outside of the frame. The recorded movements belonged to three categories: intransitive (“conversational”), transitive (simulated tool use) gestures, and meaningless hand movements, with 12 videos in each category. The list of all 24 meaningful gestures can be found in the Appendix. The movies were recorded with BENQ DC C1060 camera located 1.6 meters in front of the actor.

In Experiment 1, the to-be-categorized hand movements shown in the videos were primed by: (1) pictures of hands in postures that were most characteristic for intransitive gestures used in this study, (2) pictures of tools whose usage was pantomimed in the clips, or (3) by meaningless pictures (obtained with a polar-coordinate filter distorting the images from the intransitive and transitive categories to make them unrecognizable; e.g., in Photoshop: go to Filter, choose Distort, then Polar Coordinates, then Rectangular to Polar. For many objects the function had to be used at least twice to make them beyond recognition.). Due to differences in typical orientations of hand postures and objects, and/or sizes of objects, the pictures projected either 4 × 4 or 3 × 3.5° of visual angle (with the different sizes of priming stimuli distributed equally across the three categories of trials, and their center of gravity kept in the same spot). The stimuli were shown on a gray background (RGB 250/250/250), and were preceded by a fixation point, i.e., a black cross in the middle of the screen.

**Figure 1:**
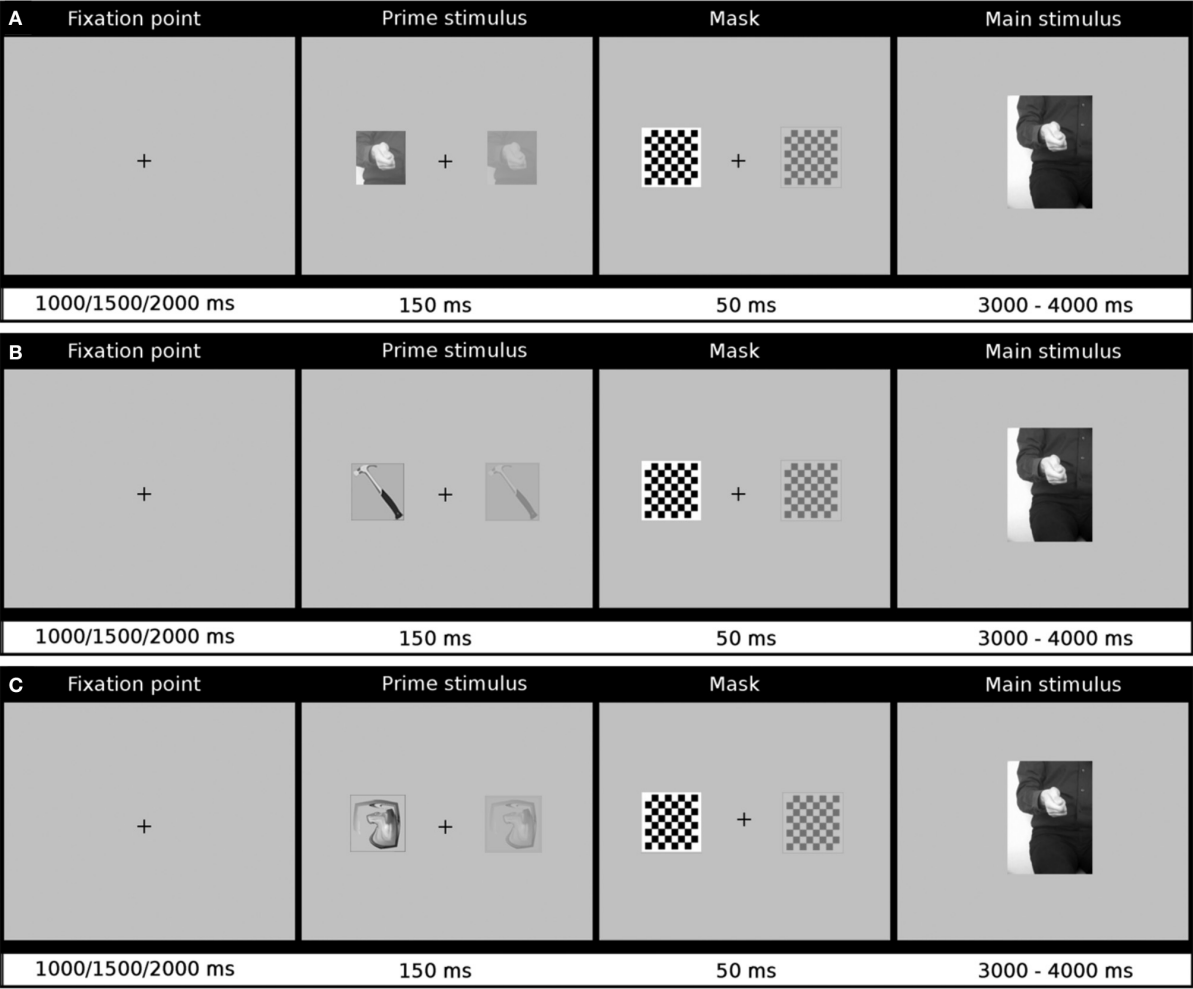
**Trial structure and timing**. In **(A)** the priming stimulus was a characteristic hand posture, in **(B)** it was a picture of a tool, and in **(C)** an image of a distorted, unrecognizable object. After a fixation point presented on a blank screen for a variable time interval (1000, 1500, or 2000 ms), the priming stimulus was shown either on the left or right (as shown by a grayed inset) for 150 ms, followed by a 50-ms mask, and a centrally presented gesture. The movie stayed on the screen until a participant responded or for up to 4 s. Additional 1-s delay interval was introduced after a movie disappeared.

The trial structure was as follows: the fixation cross alone was shown for a variable interval of 1000, 1500, or 2000 ms (thus introducing some uncertainty about the timing of the following events). Then, a prime stimulus appeared for 150 ms either on the left or right side of the screen (ca. 7° of visual angle from the fixation point), and was immediately masked for 50 ms with a checkerboard pattern, which always subtended the visual angle of 4 × 4°. (All the volunteers were explicitly instructed to maintain fixation on a central cross even if there is an additional stimulus briefly shown to the right or left of the cross.) Subsequently a movie was presented and it remained on the screen until a response was provided or for up to 4 s. Participants were asked for categorization of the watched gestures as representing: “conversation” (intransitive), “tool” (transitive), or “nonsense” (meaningless gesture). If the answer was not given (by pushing an appropriate button), the next trial started 1 s after the end of the video. The trial structure is depicted in Figure 1.

The target videos were preceded either by congruent or incongruent primes. In the former case, the prime belonged to the same category as the video (e.g., the characteristic hitch-hiking hand posture preceded the hitch-hiking gesture, a picture of a hammer preceded a movie showing a pounding gesture, or an unrecognizable image preceded a meaningless movement). In the case of incongruent primes, the stimuli belonged to a category different from the main stimulus (e.g., a picture of a hammer shown before the hitch-hiking or a meaningless gesture). Each movie appeared only three times during the course of a given experiment, and it was preceded by a prime belonging to each category. For that reason, half of the randomly selected movies in a given category were preceded by congruent primes on the left, and the remaining half by congruent primes on the right. In the case of incongruent primes—which now belonged to two different categories—for half of the randomly selected movies all the randomly assigned incongruent primes appeared on the left, and for the remaining half of the movies all the assigned primes appeared on the right. By doing this we made sure that prime location was also properly counterbalanced in each participant (not for each movie, but definitely for the whole category of movies). The order of trials was randomized differently for each participant and it was divided into three blocks, 36 trials each. There was an optional break after block one, and a compulsory break (lasting at least 1 min) after block two.

The design was implemented in SuperLab ver. 4.5.2 (Cedrus®, San Pedro, CA), and carried out with the use of Dell *Latitude D620* PC. “RB-530” response pad by Cedrus was used for measuring accuracy and response times. The patterns of responses were counterbalanced across hands and gesture types. Namely, when the left button of the pad was pressed with the left-hand fingers for intransitive gestures, the button on the right was pressed with the right-hand fingers for transitive gestures, and vice versa. For meaningless gestures participants always pressed the middle button with either their right- or left-hand fingers.

All the collected data were analyzed with two separate repeated-measures *Analyses of Variance* (*ANOVAs*), one for accuracy and one for response times to correctly categorized gestures. The within-subject factors were *gesture* (intransitive, transitive, meaningless), *prime location* (left, right), and *prime type* (congruent, incongruent). The adopted level of significance was *p* < 0.05. If necessary, the required *post-hoc* tests were Bonferroni corrected (marked as Bf-p). For reaction times accompanying a correct categorization of movies, outliers greater than two standard deviations above or below the mean (calculated across conditions, less than 1% of all trials) were removed.

#### Results

***Recognition accuracy***. Because none of the differences between the categorization accuracy for intransitive and transitive gestures was significant (neither in the main effects nor the interactions, often even without the necessary Bonferroni correction), these data will not be discussed at length here. Except for the main effect of gesture [*F*_(2, 28)_ = 8.1, *p* < 0.01; Partial Eta Squared (_p_η^2^) = 0.37; observed power (alpha) = 0.94] such that both intransitive and transitive gestures were categorized with significantly greater accuracy than meaningless hand movements (Bf-*p* < 0.01, and Bf-*p* < 0.05, respectively), all the remaining significant main effects and interactions were driven by differences in the categorization of meaningless actions. (These are of no particular interest in the absence of significant differences between the two meaningful gesture categories.) The average categorization accuracy for intransitive gestures was 83% (*SE* = 2%), for transitive gestures it was 85% (*SE* = 2.8%), and for meaningless hand movements it was only 73% (*SE* = 1.7%).

**Figure 2:**
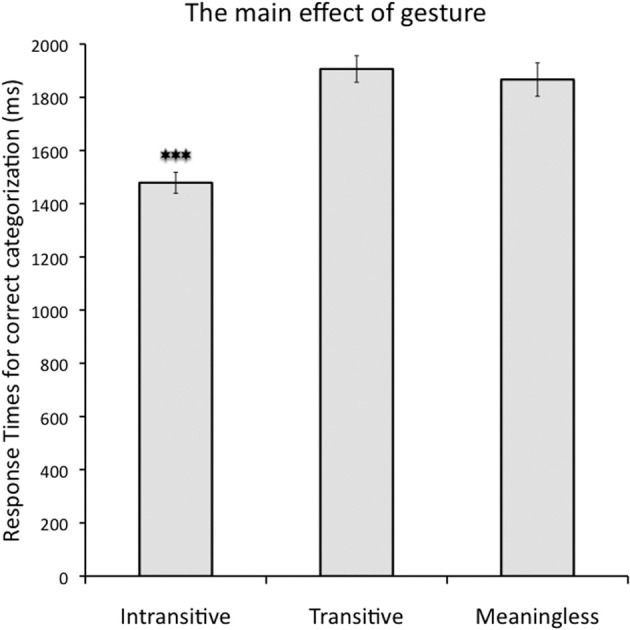
**The main effect of gesture for correctly categorized movies when pictures were used as primes**. Intransitive gestures were categorized significantly faster than transitive and meaningless movements. Response times for the latter two did not differ between each other. Asterisks indicate a difference with Bonferroni-corrected *p*-value of 0.001 (^***^).

***Response Times (RTs) for correctly categorized gestures***. There was a main effect of *gesture* [*F*_(2, 28)_ = 96.6, *p* < 0.001; _p_η^2^ = 0.87; alpha = 1.0] such that intransitive gestures were categorized significantly faster than the other two types of hand movements (mean RT for intransitive = 1479 ms, *SE* = 40 ms; transitive = 1906 ms, *SE* = 50 ms; and meaningless = 1867 ms, *SE* = 63 ms; Bf-*p* < 0.001 in both cases). No significant difference was observed between transitive and meaningless gestures (uncorrected *p* = 0.8). This effect is shown in Figure 2. There was also a main effect of *prime type* [*F*_(1, 14)_ = 16.1, *p* < 0.001; _p_η^2^ = 0.53; alpha = 0.96] such that movies preceded by a congruent prime were categorized significantly faster than on incongruent trials (mean RT for congruent = 1727 ms, *SE* = 49 ms vs. incongruent = 1775 ms, *SE* = 47 ms). The effect of prime location was not significant [*F*_(1, 14)_ = 0.23, *p* = 0.6; _p_η^2^ = 0.02; alpha = 0.07].

**Figure 3:**
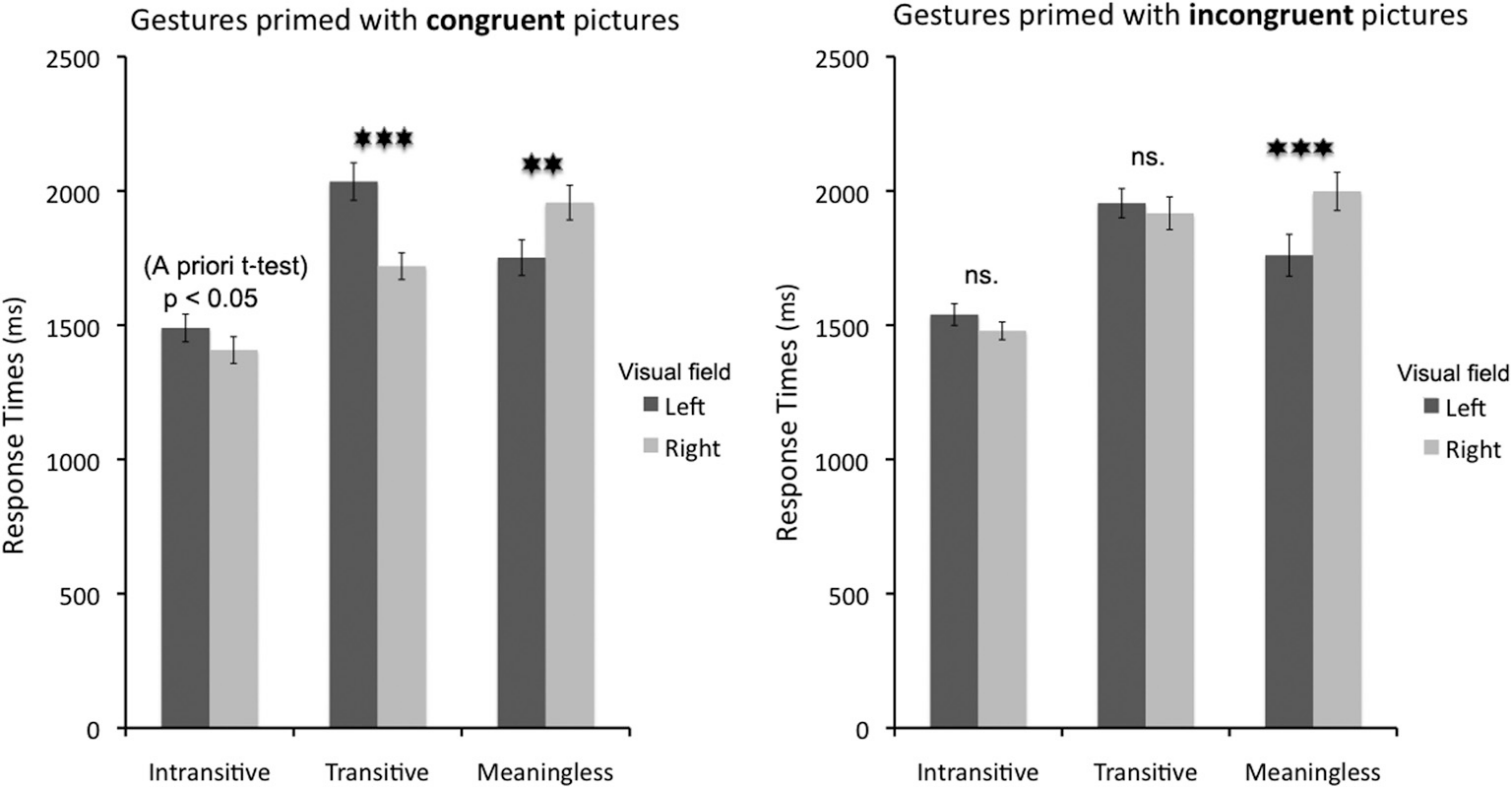
**Response times to correctly categorized intransitive gestures, tool use pantomimes, and meaningless hand movements primed by congruent or incongruent pictorial cues presented in the right or left visual field**. Transitive gestures were greatly facilitated by congruent pictorial cues on the right, and intransitive gestures showed a similar trend. The effect for meaningless movements was reversed. Incongruent pictorial cues had no effect on categorization of both meaningful gesture types, but the effect for meaningless movements was in the same direction as before. Asterisks indicate differences with Bonferroni-corrected *p*-values of at least 0.01 (^**^), or 0.001 (^***^).

Importantly, there was a significant interaction between *gesture* and *prime location* [*F*_(2, 28)_ = 37.8, *p* < 0.001; _p_η^2^ = 0.73; alpha = 1.0], such that intransitive and transitive gestures were categorized significantly faster when they were primed by a stimulus in the right visual half field (VHF), as compared to the left VHF (Bf-*p* < 0.01 and Bf-*p* < 0.001, respectively). For meaningless gestures the effect was reversed (Bf-*p* < 0.001). There was also a significant interaction between *prime location* and *prime type* [*F*_(1, 14)_ = 7.5, *p* < 0.01; _p_η^2^ = 0.35; alpha = 0.72], but the effect of congruent primes leading to faster categorization only when they were presented in the right VHF turned out to be insignificant after the Bonferroni correction (Bf-*p* = 0.06). Left-sided priming had an even weaker effect in this interaction (uncorrected *p* = 0.07). Nevertheless, all these results should be interpreted with caution because there was also a very intuitive and significant three-way interaction [between *gesture*, *prime side*, and *prime type*; *F*_(2, 28)_ = 5.3, *p* < 0.01; _p_η ^2^ = 0.28; alpha = 0.80]. Follow up tests of simple main effects were used to clarify the straightforward relations of these factors. The tests revealed that intransitive gestures tended to be categorized faster when preceded by congruent primes on the right, but this effect did not survive Bonferroni correction for multiple comparisons. Nevertheless, the impact of right-sided congruent priming on their categorization was revealed by a planned *a priori* *t*-test [*t*_(14)_ = 2.6; *p* < 0.05]. The effect of incongruent primes on the categorization of intransitive gestures was even weaker (Bf-*p* = 0.2). A significant facilitation of response times by congruent right VHF primes was observed for transitive gestures (Bf-*p* < 0.001), but not for incongruent primes (uncorrected *p* = 0.44). A completely reversed effect of primes, i.e., response facilitation when they were presented in the left VHF, and more importantly, regardless of their congruency, was observed for meaningless gestures (Bf-*p* < 0.01, and Bf-*p* < 0.001, respectively, on congruent and incongruent trials). These effects are shown in Figure 3. The mean response times, as well as mean accuracy data, for all the conditions are listed in Table 1.

**Table 1:** **Pictures as primes—Experiment 1**.

**Trial type**			**Response time (ms)**	**St. error**	**Accuracy (%)**	**St. error**	***N***
Intransitive	Left	Congruent	1490	51	77.3	3.3	15
		Incongruent	1540	40	87.4	3.0	15
	Right	Congruent	1407	49	87.5	3.9	15
		Incongruent	1479	32	80.3	2.6	15
Transitive	Left	Congruent	2035	69	90.5	3.3	15
		Incongruent	1954	54	87.6	3.2	15
	Right	Congruent	1720	49	73.3	4.2	15
		Incongruent	1917	61	86.3	3.9	15
Meaningless	Left	Congruent	1751	66	61.6	4.0	15
		Incongruent	1760	77	96.0	1.9	15
	Right	Congruent	1956	64	54.9	2.9	15
		Incongruent	1998	71	77.3	4.6	15

*Gesture type (intransitive, transitive, meaningless), prime location (left, right), prime type (congruent, incongruent) with their mean response times (ms), accuracy (%), and their standard errors of the means, for Experiment 1 with pictures serving as primes are listed*.

#### Discussion of experiment 1

Intransitive (“*conversational*,” non-object related) gestures were categorized significantly faster than transitive (simulated tool use) gestures and meaningless hand movements, whose categorization efficacy—in terms of response times—did not differ between each other. Such an outcome is consistent with an earlier observation that, at least under time pressure, healthy individuals also perform poorer during imitation of transitive actions, irrespective of whether they are meaningful or meaningless (Carmo and Rumiati, 2009). Slower performance with transitive, as well as slower and poorer categorization of meaningless gestures in our study, is therefore consistent with an idea that tool use pantomimes and nonsense hand movements, perhaps mainly due to greater movement complexity, are harder to process than more familiar intransitive gestures (Vingerhoets, 2008; Kroliczak and Frey, 2009; Króliczak, 2013b; see also Johnson-Frey et al., 2005 and Villarreal et al., 2008, where meaningless hand movements were used in a control condition). It must be emphasized, though, that the pattern of response times for correctly categorized gestures observed in our study is exactly the opposite of what was found in an fMRI report by Villarreal et al. (2008), where both the recognition of transitive and meaningless actions was significantly faster (uncorrected for multiple comparisons) than the recognition of movements belonging to an intransitive category. Yet, the testing paradigm they used was substantially different from ours.

One can speculate that the categorization of such well-known gestures as “*waving hello*” or “*hitchhiking*” may be rather automatic and less dependent on “contextual” pictorial cues, in contrast to meaningless movements, or even tool use gestures, which can be more difficult to decipher when seen unexpectedly. This hypothesis, combined with the issue of laterality of their representations (or processing) was tackled by analyzing the effects of *prime side* and *prime type*. Although the processing of transitive (tool use) gestures was slower, and comparable to meaningless movements, their categorization profited most from congruent pictures presented briefly on the right. Such an effect could either indicate that this gesture category is most strongly left lateralized or that it is particularly sensitive to relevant pictorial cues when they are processed in the left hemisphere. (Of course, this effect could also indicate a combination of both left-sided representations of tool use skills and their particular responsiveness to pictorial cues). The most efficiently categorized intransitive gestures, on the other hand, were not facilitated as much by congruent pictures on the right. This could be due to (1) a floor effect, such that one cannot simply get much faster with their categorization; (2) the fact that even the most characteristic hand postures depicted in the priming stimuli are more difficult to process than pictures of tools (which do a very good job of priming the categorization of transitive gestures), or (3) weaker laterality of representations mediating intransitive skills (e.g., Rapcsak et al., 1993; Dumont et al., 1999). Indeed, the idea that intransitive and transitive skills might be mediated by different mechanisms—with intransitive gestures being supported more strongly by the right hemisphere, or more bilaterally, i.e., by both hemispheres—figures rather prominently in most influential theories of praxis and inspires research and discussions on representations of praxis skills up until today (e.g., Rothi et al., 1991; Cubelli et al., 2000; Buxbaum, 2001; Króliczak, 2013b; see also Binkofski and Buxbaum, 2013).

Incidentally, neither the categorization of intransitive nor transitive gestures was influenced by incongruent primes, irrespective of their presentation side. Conversely, response times accompanying correct decisions on meaningless hand movements were facilitated by priming stimuli on the left, whether congruent or incongruent. Because postulating their explicit representations in one of the two hemispheres does not make much sense, it stands to reason that the categorization of such unskilled actions (i.e., movements which are not in a repertoire of our manual skills) may depend more on visuo-spatial abilities, and more deliberate processing, often associated with the right cerebral cortex (e.g., Kroliczak et al., 2007; cf. Kroliczak et al., 2008; see also Whitehouse and Bishop, 2009; Rossit et al., 2011). For these two reasons alone, the categorization of meaningless movements would be less affected by the meaning of the priming cues. In short, the observed response facilitation might be due to an engagement of related right hemisphere processing before the meaningless action is encountered.

The results so far are consistent with a long-standing idea that tool use skills are represented in the left hemisphere (see also Vingerhoets et al., 2009; Verma and Brysbaert, 2011; Garcea et al., 2012; Vingerhoets et al., 2012), whereas meaningless actions might be primarily or preferentially processed in the right hemisphere. (This is probably one of the reasons why meaningless actions make a good control condition in fMRI projects on gestures.) The status of intransitive or “communicative” gestures is less obvious because either they have more bilateral representations or are simply less dependent on the context in which they are encountered. The latter two ideas can be explored by changing the primes from pictorial to linguistic cues, and this is exactly what has been done in Experiment 2.

If intransitive gestures are represented more bilaterally, there should be no substantial facilitation from priming of these actions by closely related verbal cues presented in the right visual field (i.e., processed immediately by the left hemisphere). Moreover, one could even observe a significant interference in the form of slowing down of their categorization by incongruent words presented in the left visual field (i.e., engaging initially the right hemisphere).

### Experiment 2: categorization of gestures primed by words

#### Methods

These same 15 healthy volunteers [eight women, mean age = 23.0 (*SD* = 1.5) years of age] were involved in this study. The methods used were very similar to Experiment 1, except for the primes. Namely, the videos were now preceded by briefly presented (150-ms) linguistic cues, i.e., single words or brief two-word expressions most often associated with intransitive and transitive gestures (including their names, and object names), or meaningless strings of letters. Again, the priming stimuli were either congruent (i.e., belonged to the same category) or incongruent with the target gesture (i.e., represented the other two categories). All these primes were also immediately masked (for 50 ms) with a string of nine hashes (###…) which exceeded the longest of the priming cues by one symbol. The font size used both for words and non-words was 26 pt, whereas that for the mask was 28 pt. Thus, given that our priming cues consisted of 2–8 characters, they subtended the visual angle from 0.7 to 3°. The stimuli of various sizes were distributed more or less equally across all the conditions.

The relevant words or expressions related to meaningful gestures that were shown in the videos were chosen from the most frequent responses provided before the study by five student volunteers (two women) who did not participate, and were not involved in any way, in this research project.

Similarly to Experiment 1, the collected data were analyzed with two separate repeated-measures *ANOVAs*, for accuracy and for response times to correctly categorized gestures. Again, the within-subject factors were *gesture* (intransitive, transitive, meaningless), *prime location* (left, right), and *prime type* (congruent, incongruent). The adopted level of significance was *p* < 0.05 and, if necessary, *post-hoc* tests were Bonferroni corrected (Bf-p). For RTs to correctly categorized movies, outliers greater than two standard deviations above or below the mean were removed (less than 1% of all trials).

#### Results

***Recognition accuracy***. Similarly to Experiment 1, there was a main effect of gesture [*F*_(2, 28)_ = 4.8, *p* < 0.05; _p_η^2^ = 0.25; alpha = 0.75] but now it was such that only intransitive gestures were categorized with significantly greater accuracy than meaningless hand movements (Bf-*p* < 0.05), whereas the difference between transitive and meaningless actions did not reach significance level (Bf-*p* = 0.2). Intransitive and transitive gesture categorization was comparable (Bf-*p* = 1.0). There was also a counterintuitive main effect of *prime location* [*F*_(1, 14)_ = 10.0, *p* < 0.01; _p_η^2^ = 0.42; alpha = 0.84], such that gestures primed by a word in the left visual field were categorized with greater accuracy than gestures primed by a word in the right visual field, and this effect mirrors the one observed for RTs (see below). Although there was also a significant interaction between *gesture* and *prime type* [*F*_(2, 28)_ = 7.6, *p* < 0.01; _p_η^2^ = 0.35; alpha = 0.92], none of the differences between categorization accuracy for any gestures was significant when Bonferroni correction was applied. There were no other significant effects. The average categorization accuracy for intransitive gestures was 87% (*SE* = 1.9%), for transitive gestures it was 85% (*SE* = 2.5%), and for meaningless hand movements it was only 76% (*SE* = 3.1%).

**Figure 4:**
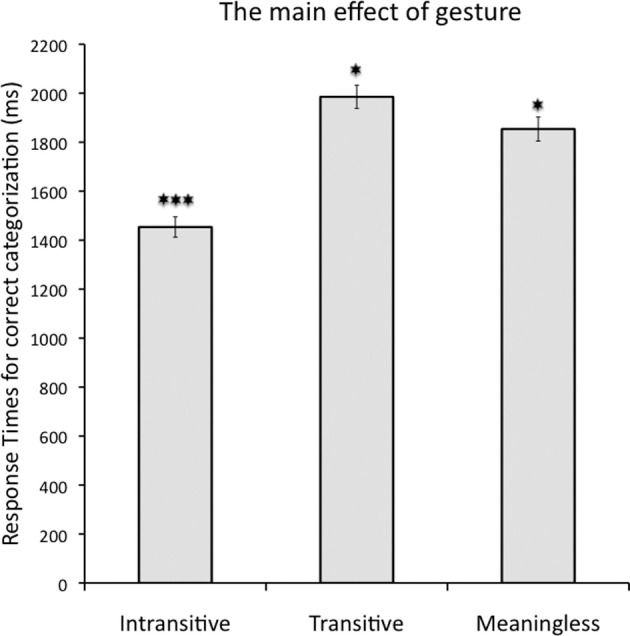
**The main effect of gesture for correctly categorized movies when words were used as primes**. Intransitive gestures were again categorized significantly faster than transitive and meaningless movements. Response times for the latter two now also differed between each other. Asterisks indicate differences with Bonferroni-corrected *p*-values of 0.05 (^*^), or 0.001 (^***^).

***RTs for correctly categorized gestures***. There was a main effect of *gesture* [*F*_(2, 28)_ = 133.0, *p* < 0.001; _p_η^2^ = 0.91; alpha = 1.0] such that similarly to Experiment 1 intransitive gestures were again categorized significantly faster (mean *RT* = 1453 ms, *SE* = 42 ms) than transitive gestures (mean *RT* = 1985 ms, *SE* = 47 ms; Bf-*p* < 0.001) and meaningless hand movements (mean *RT* = 1854 ms, *SE* = 49 ms; Bf-*p* < 0.001). Importantly, counter to Experiment 1, transitive gestures were categorized significantly slower than meaningless movements (Bf-*p* < 0.05). This effect is shown in Figure 4. In sharp contrast to Experiment 1, a main effect of *prime location* was now significant [*F*_(1, 14)_ = 27.6, *p* < 0.001; _p_η^2^ = 0.66; alpha = 0.99], but it was also quite unexpected, such that the studied gestures were categorized significantly faster when the priming stimuli were presented in the left VHF (mean *RT* = 1736 ms, *SE* = 41 ms) as compared to the right VHF (mean *RT* = 1793 ms, *SE* = 43 ms). Finally, there was also a counterintuitive main effect of *prime type* [*F*_(1, 14)_ = 6.3, *p* < 0.05; _p_η^2^ = 0.31; alpha = 0.64] with gesture categorization being significantly faster following incongruent primes (mean *RT* = 1743 ms, *SE* = 44 ms) as compared to congruent primes (mean *RT* = 1785 ms, *SE* = 40 ms). The latter two main effects should not be overrated, though, given the significant interactions that were also obtained.

**Figure 5:**
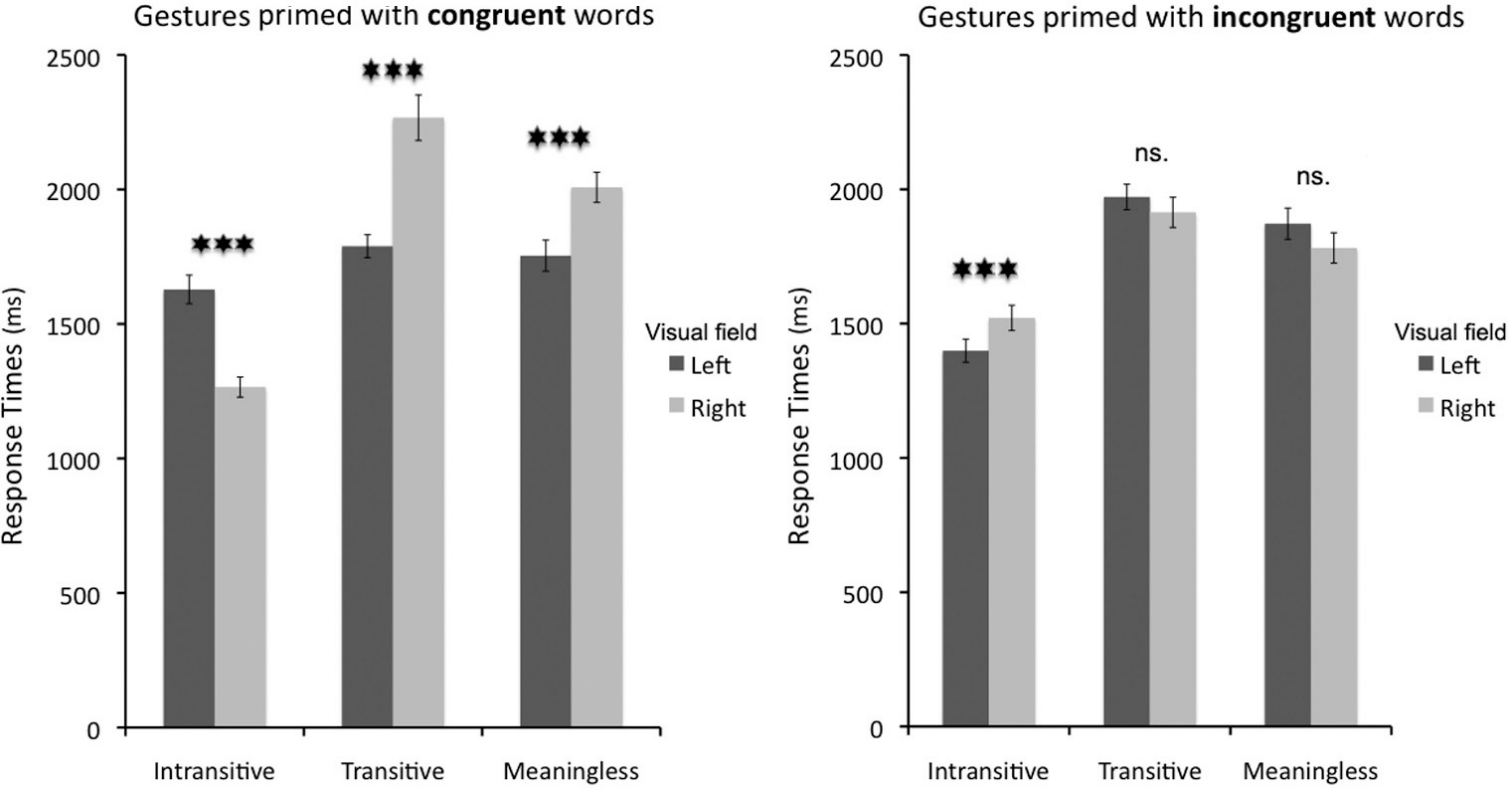
**Response times to correctly categorized intransitive gestures, tool use pantomimes, and meaningless hand movements primed by congruent or incongruent linguistic cues presented in the right or left visual field**. Intransitive gestures were greatly facilitated by congruent linguistic cues on the right, whereas transitive gestures and meaningless movements showed the opposite effect. Incongruent linguistic cues affected the categorization of only intransitive gestures. Asterisks indicate differences with Bonferroni-corrected *p*-values of at least 0.001 (^***^).

The first significant interaction was between *gesture* and *prime location* [*F*_(2, 28)_ = 18.6;, *p* < 0.001; _p_η^2^ = 0.57; alpha = 1.0]. This effect was such that intransitive gestures were categorized significantly faster following priming words on the right (Bf-*p* < 0.01), whereas transitive gestures were categorized significantly faster following priming words on the left (Bf-*p* < 0.001). The effect of prime location for meaningless hand movements was similar to transitive gestures but turned out to be insignificant after Bonferroni correction (uncorrected *p* = 0.04). There was also a significant interaction between *prime location* and *prime type* [*F*_(1, 14)_ = 12.9, *p* < 0.01; _p_η^2^ = 0.48; alpha = 0.92]. This interaction, on the other hand, suggested that gesture categorization was significantly slower when the right-sided priming words were actually congruent (Bf-*p* < 0.01), whereas the left-sided words had no effect whatsoever (uncorrected *p* = 0.37). As in Experiment 1, all the above effects, including the two 2-way interactions, should be interpreted with great caution because there was also a much more intuitive significant three-way interaction between *gesture*, *prime side*, and *prime type* [*F*_(2, 28)_ = 54.0, *p* < 0.001; _p_η ^2^ = 0.79; alpha = 1.0]. Similarly to Experiment 1, tests of simple main effects were utilized to clarify the apparently complex relationships between these factors. The tests revealed that intransitive gestures were categorized significantly faster when primed by congruent words on the right (as compared to congruent words on the left; Bf-*p* < 0.001), whereas their categorization was significantly slower when primed by incongruent words on the right (as compared to incongruent words on the left; Bf-*p* < 0.001). In sharp contrast, for both transitive gestures and meaningless hand movements the effect of *prime type* was reversed because their categorization was significantly slower when congruent cues appeared on the right (as compared to congruent cues on the left; Bf-*p* < 0.001 in both cases), whereas there was no impact of incongruent priming words on their categorization that could be related to the presentation side (uncorrected *p* = 0.19 for transitive, and *p* = 0.09 for nonsense movements). These effects are shown in Figure 5. The mean response times, as well as mean accuracy data, for all the conditions from Experiment 2 are listed in Table 2.

**Table 2:** **Words as primes—Experiment 2**.

**Trial type**			**Response time (ms)**	**St. error**	**Accuracy (%)**	**St. error**	***N***
Intransitive	Left	Congruent	1628	53	97.3	1.8	15
		Incongruent	1399	43	90.5	1.7	15
	Right	Congruent	1265	38	79.9	4.4	15
		Incongruent	1521	47	82.3	3.8	15
Transitive	Left	Congruent	1789	43	84.4	6.0	15
		Incongruent	1971	47	89.4	3.0	15
	Right	Congruent	2266	85	78.9	3.0	15
		Incongruent	1914	56	87.8	2.7	15
Meaningless	Left	Congruent	1754	58	84.4	4.1	15
		Incongruent	1872	58	73.9	3.6	15
	Right	Congruent	2008	56	75.6	3.6	15
		Incongruent	1782	56	70.1	4.4	15

*Gesture type (intransitive, transitive, meaningless), prime location (left, right), prime type (congruent, incongruent) with their mean response times (ms), accuracy (%), and their standard errors of the means, for Experiment 2 with words as primes are listed*.

**Figure 6:**
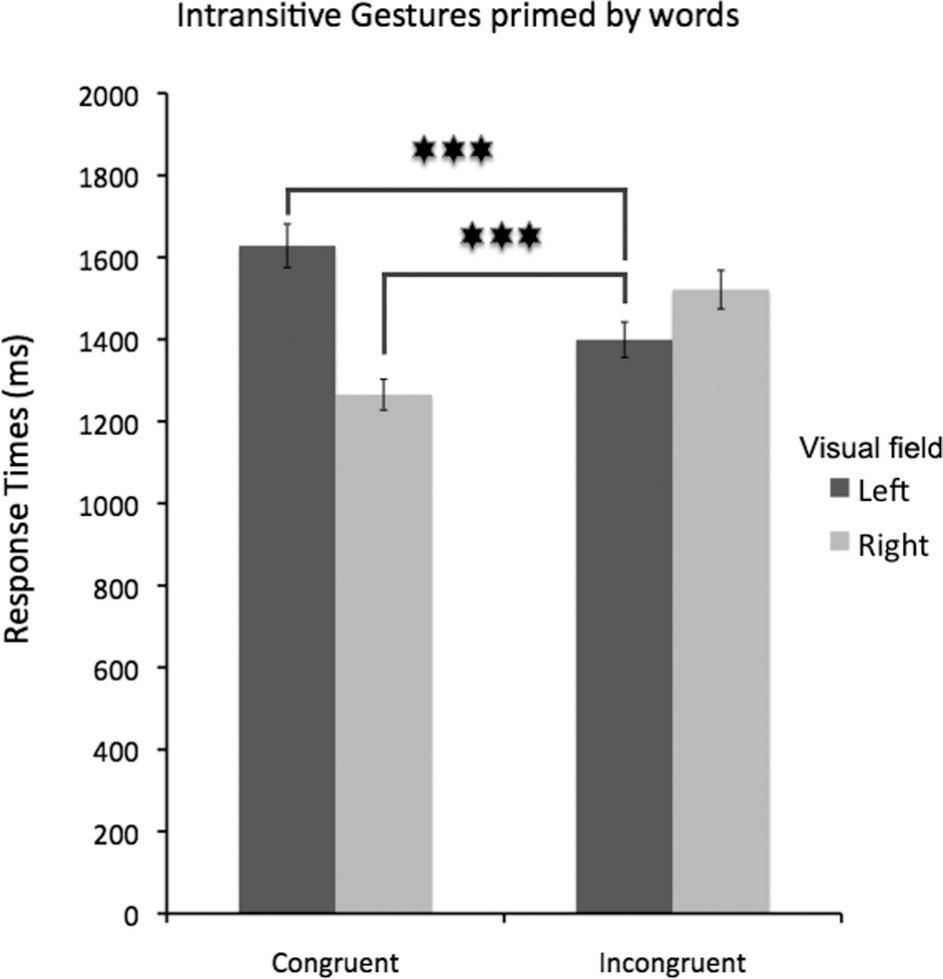
**The effects of incongruent left-sided cues on intransitive gesture categorization**. There are two important comparisons that are considered. As can be predicted, such incongruent cues on the left significantly slowed the categorization of intransitive gestures as compared to congruent cues on the right. Yet, at the same time their negative impact was weaker than that of congruent cues presented on the left. This effect is consistent with an idea that right-hemisphere processing is also important in the categorization of conventionalized intransitive gestures. Asterisks indicate differences with Bonferroni-corrected *p*-values of at least 0.001 (^***^).

Finally, for clarification of the obtained interaction effects, two additional *post-hoc* tests are described here to compare response times accompanying correct categorization of intransitive gestures following incongruent linguistic cues on the left with the effects of congruent cues on the right, and the impact of congruent cues on the left. Both of the observed differences were significant (Bf-*p* < 0.001 in both cases). Namely, although the categorization of intransitive gestures following incongruent cues on the left was significantly slower as compared to the effects of congruent cues on the right, it was at the same time significantly faster when compared to the effects of congruent cues on the left. This effect is shown in Figure 6. In other words, for the categorization of intransitive gestures, taking into account a valid cue from the left visual field requires significantly more time than a rejection of an invalid cue. (Of course, an observation that categorizing these gestures is faster following incongruent words on the left as compared to incongruent words on the right has been described in the previous paragraph, with an emphasis on the effect that incongruent cues on the right slowed participants' responses.)

#### Discussion of experiment 2

Because for categorization accuracy none of the differences between intransitive and transitive gestures was significant, in the discussion we will again focus only on response time results. It should be noted, though, that comparable accuracy in the two conditions indicates that both of the meaningful gesture categories have fine-grained representations in the brain, and the retrieval of these representations cannot be easily interfered with. In healthy participants, the differences in access and/or interference effects are only or primarily apparent when response times are analyzed, although in patients differences in accuracy following left- or right-sided lesions are often quite clear (Roy et al., 1991; Foundas et al., 1999; Haaland et al., 2000; cf. Rapcsak et al., 1993; Dumont et al., 1999; Mozaz et al., 2002, see also Stamenova et al., 2010).

Similarly to Experiment 1, intransitive (“conversational”) gestures were again categorized significantly faster than transitive (tool use) pantomimes and meaningless hand movements. Yet, this time the categorization efficacy also differed between the latter two, with correct responses to transitive gestures being significantly slower than to meaningless actions. As noted above, the better performance with intransitive gestures is quite consistent with an earlier report on differences in accuracy observed during imitation of the two gesture categories under time constraints (Carmo and Rumiati, 2009). Indeed, these and our current results, as well as the outcomes of other behavioral (e.g., Mozaz et al., 2009) and recent neuroimaging studies on intransitive and transitive gestures support a view that transitive actions, as belonging to a less familiar category, rather than being differently represented—i.e., more left lateralized—are more difficult to process and/or perform [Kroliczak and Frey, 2009; Króliczak, 2013b; but cf. behavioral and neuroimaging results of Villarreal et al. (2008); see also Stamenova et al., 2010]. Interestingly, in the context of Experiment 2, this can be also said when their categorization is compared with that of actions deprived of meaning. Namely, meaningless actions were also categorized with greater ease than transitive gestures. Although meaningless actions were also categorized with lowest accuracy, speed-accuracy trade-off cannot be the major factor involved and its effect must have been combined with a greater adverse impact of linguistic cues on the categorization of transitive gestures.

As to the impact of these laterally presented cues in the form of words or short phrases on categorization, all the three gesture types were affected by congruent primes presented on the right. Yet it was only the categorization of intransitive gestures which—counter to a hypothesis of their more bilateral representations—was greatly facilitated, whereas the processing of transitive and meaningless actions was substantially hindered (as compared to the left-sided cues of the same kind). Conversely, the impact of incongruent linguistic cues was observed only for the intransitive gesture category, and it was actually the opposite of what was found for congruent primes. Namely, whereas right-sided congruent linguistic cues have facilitated performance, incongruent primes presented on the right have now slowed down the categorization of intransitive gestures.

Even though the latter finding is consistent with a view that the representations of intransitive gestures—similarly to language skills, with which they must be closely related (Kroliczak et al., 2011)—are strongly left lateralized, this interpretation should be exercised with caution. After all, although the verification of an incongruent linguistic cue from the left visual field, i.e., processed first in the right hemisphere, takes significantly longer than the evaluation of a congruent cue in the left hemisphere, nevertheless, and quite surprisingly such verification takes significantly less time than the processing of a congruent cue in the right hemisphere. This is not what would be expected if the representations of intransitive gestures were exclusively left lateralized.

The above-mentioned counterintuitive sensitivity to right hemisphere processing further suggests that intransitive gestures are represented somewhat differently from transitive pantomimes. The latter, conversely to intransitive but similarly to meaningless movements, were adversely affected by congruent linguistic cues presented on the right (i.e., projected to the left hemisphere). This could be due to the fact that some of its processing is either incompatible with, or perhaps engages excessively, the mechanisms to be also involved in their categorization. A different kind of representation for tool use gestures is also suggested by the lack of sensitivity to irrelevant linguistic cues (cf. Kroliczak et al., 2006), irrespective of the presentation side.

## General comments

Consistently with earlier reports (Carmo and Rumiati, 2009; Kroliczak and Frey, 2009; Króliczak, 2013b), this study provides further evidence supporting an idea that intransitive gestures as less complex, highly conventionalized, and for that reason more often seen and used in naturalistic settings, are also easier to categorize as compared to rarely perceived and performed transitive gestures (tool use pantomimes), as well as meaningless hand movements. This is the case regardless of the testing conditions. Moreover, in the context of additional pictorial cues (i.e., images of hand postures for intransitive, and tools for transitive gestures), the response facilitation observed for both gesture categories was in the same direction, thus implying the involvement of some common mechanisms. Even though transitive gestures, perhaps as more difficult to retrieve in the first place, gained way more from these “prompts,” as such, our results from a study using pictures as primes do not undermine a view that the two gesture categories might be processed within a common network. After all, the finding that the categorization of both gesture types (though much less in the case of intransitive gestures) was more efficient when the relevant pictorial cues were presented on the right—i.e., projected to the left hemisphere—is yet another piece of evidence that the understanding and control of meaningful gesture depends to a high degree on left-lateralized representations of praxis skills. It should be emphasized still again, though, that intransitive gestures depend substantially less on their input. (Yet, they are easier anyways.)

Consistent with the observation that intransitive gestures may be somewhat less lateralized—or rather more bilaterally represented—is our second major finding, namely that of their particular sensitivity to linguistic cues processed in both hemispheres. On the one hand, a dramatic facilitation in categorizing them as “conversational” following right-sided words or phrases supports the view of their dependence on left-lateralized mechanisms, which might be common with language functions (cf. Kroliczak et al., 2011; Vingerhoets et al., 2013; see also Goldenberg, 2013b; Króliczak, 2013a). On the other hand, although not surprisingly their categorization is substantially slower when relevant cues are first processed in the right hemisphere, this processing is in fact more detrimental than a verification that a right-sided cue is irrelevant. These findings are in fact consistent with a very long-standing conviction (e.g., Morlass, 1928) that the ability to perform and understand conventionalized (intransitive) gestures, while relying on general praxis representations, may also call for mechanisms and skills (e.g., social knowledge) implemented in different brain areas, including the right hemisphere. As suggested in the Introduction, this idea has indeed prominently figured in modern theories of praxis (cf. Gonzalez Rothi et al., 1991) implying that the mechanisms involved in retrieval of intransitive actions (including manual emblems) may be distributed across both hemispheres.

Finally, and quite unexpectedly, transitive gestures do not show much affinity to relevant linguistic cues, since their categorization was much slower in their presence and resembled that of meaningless actions. Yet, one cannot judge from such data that their representations are not left lateralized.

## Limitations of the study

It would be best if eye movements were monitored in such a paradigm, although prime duration of only 150 ms and the immediate mask make it less of a problem. It would be also better if primes appeared simultaneously in the right and left visual field, and attention to these lateralized primes was directly controlled for by an additional central cue. (For any further suggestions on and/or criticisms of visual half-field paradigms, see Hunter and Brysbaert, 2008).

## Conclusions

In sum, this study shows evidence that the categorization of intransitive gestures may also draw on contributions from processes or mechanisms taking place outside of the left-lateralized praxis representation network. Indeed, it is justified to say that some of these processes (or mechanisms) might be located in the right cerebral hemisphere. Furthermore, and quite surprisingly, this seems to be particularly true when linguistic processing is involved. Yet, this conclusion would be much stronger were it not for the fact that such linguistic cues also affect the processing of tool use gestures in a rather unexpected way.

## Appendix: meaningful stimulus videos

*Intransitive gestures*: Beckoning, Counting, Flicking, Hitchhiking, Pointing, Scolding, Shooing, Snapping, Stopping, Talking, Wavering, Waving.

*Transitive gestures*: Dialing, Painting, Pounding, Pouring, Reeling, Scrubbing, Sewing, Typing, Unlocking, Using a remote control, Using a spoon, Writing.

## Author contributions

This project was conceptualized by Honorata Helon and Gregory Króliczak. Data was collected by Honorata Helon, and analyzed by Gregory Króliczak and Honorata Helon. The manuscript was written by Gregory Króliczak and Honorata Helon.

### Conflict of interest statement

The authors declare that the research was conducted in the absence of any commercial or financial relationships that could be construed as a potential conflict of interest.

## References

[B1] Binkofski F. Buxbaum L. J. (2013). Two action systems in the human brain. Brain Lang. 127, 222–229 10.1016/j.bandl.2012.07.007 22889467 PMC4311762

[B2] Buxbaum L. J. (2001). Ideomotor apraxia: a call to action. Neurocase 7, 445–458 10.1093/neucas/7.6.445 11788737

[B3] Carmo J. C. Rumiati R. I. (2009). Imitation of transitive and intransitive actions in healthy individuals. Brain Cogn. 69, 460–464 10.1016/j.bandc.2008.09.007 18976850

[B4] Cubelli R. Marchetti C. Boscolo G. Della Sala S. (2000). Cognition in action: testing a model of limb apraxia. Brain Cogn. 44, 144–165 10.1006/brcg.2000.1226 11041987

[B5] Dumont C. Ska B. Schiavetto A. (1999). Selective impairment of transitive gestures: an unusual case of apraxia. Neurocase 5, 447–458 10.1080/13554799908402739

[B6] Foundas A. L. Macauley B. L. Raymer A. M. Maher L. M. Rothi L. J. Heilman K. M. (1999). Ideomotor apraxia in Alzheimer disease and left hemisphere stroke: limb transitive and intransitive movements. Neuropsychiatry Neuropsychol. Behav. Neurol. 12, 161–166 10456799

[B7] Frey S. H. (2008). Tool use, communicative gesture and cerebral asymmetries in the modern human brain. Philos. Trans. R. Soc. Lond. B Biol. Sci. 363, 1951–1957 10.1098/rstb.2008.0008 18292060 PMC2606701

[B8] Garcea F. E. Almeida J. Mahon B. Z. (2012). A right visual field advantage for visual processing of manipulable objects. Cogn. Affect. Behav. Neurosci. 12, 813–825 10.3758/s13415-012-0106-x 22864955 PMC3508329

[B9] Goldenberg G. (2013a). Apraxia. WIREs. Cogn. Sci. 4, 453–462 10.1002/wcs.1241 26304239

[B10] Goldenberg G. (2013b). Apraxia in left-handers. Brain 136, 2592–2601 10.1093/brain/awt181 23864275

[B11] Gonzalez Rothi L. J. Ochipa C. Heilman K. M. (1991). A cognitive neuropsychological model of limb praxis. Cogn. Neuropsychol. 8, 443–458 10.1080/02643299108253382

[B12] Haaland K. Y. Harrington D. L. Knight R. T. (2000). Neural representations of skilled movement. Brain 123, 2306–2313 10.1093/brain/123.11.2306 11050030

[B13] Hunter Z. R. Brysbaert M. (2008). Visual half-field experiments are a good measure of cerebral language dominance if used properly: evidence from fMRI. Neuropsychologia 46, 316–325 10.1016/j.neuropsychologia.2007.07.007 17716695

[B14] Johnson-Frey S. H. Newman-Norlund R. Grafton S. T. (2005). A distributed left hemisphere network active during planning of everyday tool use skills. Cereb. Cortex 15, 681–695 10.1093/cercor/bhh169 15342430 PMC1364509

[B15] Króliczak G. (2013a). Praxis in left-handers. Kultura i Edukacja (Cult. Educ.) 6, 5–31

[B16] Króliczak G. (2013b). Representations of transitive and intransitive gestures: perception and imitation. J. Neurosci. Neuroeng. 2, 195–210 10.1166/jnsne.2013.1050

[B17] Kroliczak G. Cavina-Pratesi C. Goodman D. A. Culham J. C. (2007). What does the brain do when you fake it? An fMRI study of pantomimed and real grasping. J. Neurophysiol. 97, 2410–2422 10.1152/jn.00778.2006 17229828

[B18] Kroliczak G. Frey S. H. (2009). A common network in the left cerebral hemisphere represents planning of tool use pantomimes and familiar intransitive gestures at the hand-independent level. Cereb. Cortex 19, 2396–2410 10.1093/cercor/bhn261 19181695 PMC2742597

[B19] Kroliczak G. McAdam T. D. Quinlan D. J. Culham J. C. (2008). The human dorsal stream adapts to real actions and 3D shape processing: a functional magnetic resonance imaging study. J. Neurophysiol. 100, 2627–2639 10.1152/jn.01376.2007 18768646

[B20] Kroliczak G. Piper B. J. Frey S. H. (2011). Atypical lateralization of language predicts cerebral asymmetries in parietal gesture representations. Neuropsychologia 49, 1698–1702 10.1016/j.neuropsychologia.2011.02.044 21382390 PMC3100506

[B21] Kroliczak G. Westwood D. A. Goodale M. A. (2006). Differential effects of advance semantic cues on grasping, naming, and manual estimation. Exp. Brain Res. 175, 139–152 10.1007/s00221-006-0524-5 16733705

[B22] Morlass J. (1928). Contribution à l'Etude de l'Apraxie. Paris: Amédee Legrand

[B23] Mozaz M. Rothi L. J. Anderson J. M. Crucian G. P. Heilman K. M. (2002). Postural knowledge of transitive pantomimes and intransitive gestures. J. Int. Neuropsychol. Soc. 8, 958–962 10.1017/S1355617702870114 12405548

[B24] Mozaz M. J. Crucian G. P. Heilman K. M. (2009). Age-related changes in arm-hand postural knowledge. Cogn. Neuropsychol. 26, 675–684 10.1080/02643291003651571 20401769

[B25] Rapcsak S. Z. Ochipa C. Beeson P. M. Rubens A. B. (1993). Praxis and the right hemisphere. Brain Cogn. 23, 181–202 10.1006/brcg.1993.1054 8292325

[B26] Rossit S. Malhotra P. Muir K. Reeves I. Duncan G. Harvey M. (2011). The role of right temporal lobe structures in off-line action: evidence from lesion-behavior mapping in stroke patients. Cereb. Cortex 21, 2751–2761 10.1093/cercor/bhr073 21508302

[B27] Rothi L. J. G. Ochipa C. Heilman K. M. (1991). A cognitive neuropsychological model of limb praxis. Cogn. Neuropsychol. 8, 443–458 10.1080/02643299108253382

[B28] Roy E. A. Square-Storer P. Hogg S. Adams S. (1991). Analysis of task demands in apraxia. Int. J. Neurosci. 56, 177–186 10.3109/00207459108985414 1938133

[B29] Rumiati R. I. Papeo L. Corradi-Dell'Acqua C. (2010). Higher-level motor processes. Ann. N.Y. Acad. Sci. 1191, 219–241 10.1111/j.1749-6632.2010.05442.x 20392283

[B30] Stamenova V. Roy E. A. Black S. E. (2010). Associations and dissociations of transitive and intransitive gestures in left and right hemisphere stroke patients. Brain Cogn. 72, 483–490 10.1016/j.bandc.2010.01.004 20167414

[B31] Verma A. Brysbaert M. (2011). A right visual field advantage for tool-recognition in the visual half-field paradigm. Neuropsychologia 49, 2342–2348 10.1016/j.neuropsychologia.2011.04.007 21527265

[B32] Villarreal M. Fridman E. A. Amengual A. Falasco G. Gerscovich E. R. Ulloa E. R. (2008). The neural substrate of gesture recognition. Neuropsychologia 46, 2371–2382 10.1016/j.neuropsychologia.2008.03.004 18433807

[B33] Vingerhoets G. (2008). Knowing about tools: neural correlates of tool familiarity and experience. Neuroimage 40, 1380–1391 10.1016/j.neuroimage.2007.12.058 18280753

[B34] Vingerhoets G. Acke F. Alderweireldt A. S. Nys J. Vandemaele P. Achten E. (2012). Cerebral lateralization of praxis in right- and left-handedness: same pattern, different strength. Hum. Brain Mapp. 33, 763–777 10.1002/hbm.21247 21500314 PMC6870330

[B35] Vingerhoets G. Acke F. Vandemaele P. Achten E. (2009). Tool responsive regions in the posterior parietal cortex: effect of differences in motor goal and target object during imagined transitive movements. Neuroimage 47, 1832–1843 10.1016/j.neuroimage.2009.05.100 19523524

[B36] Vingerhoets G. Alderweireldt A. S. Vandemaele P. Cai Q. Van der Haegen L. Brysbaert M. (2013). Praxis and language are linked: evidence from co-lateralization in individuals with atypical language dominance. Cortex 49, 172–183 10.1016/j.cortex.2011.11.003 22172977

[B37] Whitehouse A. J. Bishop D. V. (2009). Hemispheric division of function is the result of independent probabilistic biases. Neuropsychologia 47, 1938–1943 10.1016/j.neuropsychologia.2009.03.005 19428426 PMC2706326

